# Impact of mannose-binding lectin gene polymorphism on lung functions among workers exposed to airborne *Aspergillus* in a wastewater treatment plant in Egypt

**DOI:** 10.1007/s11356-022-20234-w

**Published:** 2022-04-22

**Authors:** Amal Saad-Hussein, Gehan Moubarz, Heba Mahdy-Abdallah, Mona Adel Helmy

**Affiliations:** Environmental and Occupational Medicine Department, National Research Centre, Environment and Climate Change Research Institute, Cairo, Egypt

**Keywords:** Wastewater treatment plant’s workers, *Aspergillus* sp., Pulmonary function tests, MBL2 gene polymorphism, Serum MBL

## Abstract

In this study, the risk of *Aspergillus* (*Asp.*) positivity and its respiratory health impacts on wastewater treatment plant (WWTP) workers were studied. In addition, it identified the geno-susceptibility role of mannose-binding lectin 2 (MBL2) gene polymorphisms and the mannose-binding lectin (MBL) serum levels on the pulmonary functions of the *Asp.-*positive workers. Pulmonary function tests (PFTs) were performed for 89 workers from a selected WWTP, after exclusion of the smokers. Molecular identification of *Asp.* blood positivity was done by 18S rRNA sequencing. Determination of MBL2 gene polymorphism and estimation of MBL serum levels were done. PFTs revealed abnormalities in 49.2% of the workers. *Asp.* was positive in 42.5% of the workers with different species. Among the *Asp.*-positive workers, 6.5% of the workers were with obstructive PFTs, 12.9% with restriction, and 22.6% with combined PFT abnormalities. MBL2 genotyping showed that wild genotype AA was common (68.5%) among *Asp.-*positive workers compared to the other genotypes. This allele, whether homozygous or heterozygous, was significantly associated with decline in PFTs of the exposed workers. MBL serum levels were significantly lower in workers with obstructive, restrictive, and combined PFT abnormalities compared to those with normal PFTs, and in the workers with *Asp.*-positive species than the *Asp.*-negative workers. Moreover, it was significantly lower in workers with *Asp. fumigatus* compared to that in the workers with other *Asp.* species, and in the *Asp.*-positive workers with homozygous or heterozygous A allele compared to that in the *Asp.*-positive workers with homozygous B allele. Working in a WWTP can be associated with impaired PFTs due to exposure to airborne fungi. MBL2 genotyping showed that *Asp.-*positive workers with homozygous or heterozygous A allele were at risk to develop decline in their PFTs.

## Introduction

Respiratory health problems are frequently associated with occupational exposure to fungi that are mostly associated with worsening in the lung functions of the exposed workers (Minárik et al. 2020; Eduard 2021). Spores and mycelium fragments of fungi may be present as single particles or complex aggregate which adhere to dust particles, and exposure to those aggregated particles may present a risk that depends on the health status of the workers (Sabino et al. 2019). The guidelines of the World Health Organization (WHO) reported that occupants of damp indoor environments are at risk of upper and lower respiratory symptoms, asthma, respiratory infections, pneumonitis, allergic rhinitis, and bronchitis (Mendell et al. 2011). Airborne fungal particles found at indoor environments can be inhaled by exposed persons. Conidia of *Asp. fumigatus*, with a diameter of approximately 1 to 4 μm, are deposited in the lower respiratory tract, whereas the larger conidia of other *Asp.* species, such as *Asp. flavus* and *Asp. niger*, tend to be deposited in the paranasal sinuses and upper airways (Latge 1999; Ben-Ami et al. 2010).

Fungal infection is one of the occupational health problems in many occupational settings such as ceramic, textile, and bakeries (Saad-Hussein et al. 2006; Saad-Hussein et al. 2016; Viegas et al. 2020). The environment of waste water treatment plants (WWTPs) provides a suitable moisture condition for fungal growth (Thirumala et al. 2012). Thus, workers that handle wastewater could be exposed to high concentrations of fungal particles (Viegas et al. 2017). Numerous studies reported respiratory symptoms among workers at sewage treatment plants (Rylander 1999; Thorn and Kerekes 2001; Thorn and Beijer 2004; Muzaini et al. 2021). Swan et al. (2003) found that *Aspergillus* species was a dominant isolate in the air of WWTPs, about 35% of all identified fungi.

*Aspergillus* infections depend on the interplay between host health status and their susceptibility of defense against infections, besides the virulence of the microorganism. Mannose-binding lectin (MBL) is synthesized by the liver and binds to microbes. Low MBL can indirectly regulate the immune response against fungal pathogens, as low MBL levels are associated with an increase in the production of inflammatory cytokines, such as interleukin-6 (IL-6), IL-1-beta, and tumor necrosis factor-α. These cytokines are involved in the immune response against fungal pathogens; hence, MBL plays an important role as regulators of the innate immune responses (Huffnagle and Deepe 2003; Romani 2004). Enhanced production of IL-6, a fibrogenic cytokine, can activate fibrotic process that often leads to rapid and severe deterioration of the respiratory mechanics and gas exchange properties (Luzina et al. 2015).

Mannose-binding lectin 2 (MBL2) gene polymorphisms produce intermediate/low/null or normal MBL serum levels (Super et al. 1989). A mutation in the structurally encoding region of the MBL2 gene will lead to reduced serum levels of the functional protein MBL that could result in impaired immune clearance of *Asp.* species. MBL levels, mainly due to genetic influences, have been largely described to be associated with the susceptibility of the body to invasive infections and poor outcome (Neth et al. 2000; Eisen and Minchinton 2003).

The aim of this study was to define the risk of *Asp.* positivity in WWTP workers, and its potential hazards on their lung functions, and in addition, identify the geno-susceptibility role of the MBL2 gene and the MBL serum levels on the respiratory functions of *Asp.*-positive workers.

## Methods

A cross-section descriptive study was conducted. A wastewater treatment station in Cairo, Egypt, was selected to conduct this study. All the workers from the different sectors in the selected WWTP were included in the study (114 workers). After exclusion of the smokers, the workers included in the present study were 89 workers. It was previously reported that workers in the selected WWTP are exposed to different *Asp.* species in their working environment (Saad-Hussein 2017-2020; Saad-Hussein et al. 2022).

### Questionnaire

A written informed consent was obtained from all participants. The questionnaires were fulfilled through a personal interview. The questionnaire included personal data, smoking habit, and details of residential environment, current and previous occupational histories, and medical history of acute and chronic respiratory problems, and personal complaints related to respiratory system.

### Lung function test

A spirometry test was performed strictly in accordance with the Association of Respiratory Technician and Physiologists (ARTP) guidelines (Sylvester et al. 2020). It was done using a portable spirometer Model Smart PFT USB. Subject information including serial number, name, birth date, weight, height, and race were entered. The subject was instructed to breathe normally from 5 to 7 times. He was then asked to take deep breath slowly and then expire until the end very slowly. He was then asked to breathe at normal rate again for 5–7 times. To perform the forced expiratory function, the subject was asked to breathe normally 5–7 times. He was then asked to inspire deeply as maximum as possible, and then expire forcibly until the end of expiration as much as possible. He was then asked to breathe normally again for 5–7 times. For accuracy of the results, the test was repeated at least three times. A difference of 5% between the three results is acceptable. The best result was registered.

### Collection of blood samples and DNA extraction

About 5 ml blood was collected in sterile tubes; 3 ml was left to clot for 30 min at 37°C and then centrifuged at 3000 rpm for 10 min. The separated serum was kept frozen at −20°C until use. Another 2 ml blood was collected on an EDTA tube, and was stored at −20°C until DNA extraction was performed using Genomic DNA Purification kit (Gene JET™/Fermentas).

### Molecular identification of Aspergillus infection by 18S rRNA sequencing

18S rRNA gene region was amplified with the primers ASAP1:5^′^-CAGCGAGTACATCACCTTGG-3^′^ and ASAP2:5^′^-CCATTGTTGAAACTTTTAACTGATT3^′^. The PCR reaction mixtures (25 μl) contained 5 μl of the extracted DNA, Dream Tag Green PCR master mix(2X) 12.5 μl, 2 μl of forward primer (0.4 μM), 2 μl of reverse primer (0.4 μM), and water nuclease completed to 25 μl. For ASAP PCR reaction, each mixture was heated to 94°C for 4 min and PCR was performed with the following program for the Mastercycler (Eppendorf): 94°C for 1 min; 55°C for 2 min; and 72°C for 90s, all repeated for 30 cycles. Thermal cycling was terminated by polymerization at 72°C for 10 min. A total of 20 μl of each mixed PCR product with loading dye was used to perform electrophoresis in 1.5% (w/v) agarose gel, with 5 μl 1Kb DNA Ladder as a molecular marker in parallel. These sequences amplify an approximately 521 bp product from the 3′ end of the 18S target (Sugita et al. 2004).

The amplified products were purified using the QIA quick PCR purification kit, according to the manufacturer’s directions. The resulting DNA purified sequences were sequenced with primers for 18S rDNA by an automated DNA sequencer (ABI model 377; Applied Biosystems). The sequences of fungal isolates were then compared to those in GenBank (National Centre for Biotechnology Information; http://www.ncbi.nih.gov/) using the Basic Local Alignment Search Tool for nucleotide sequences (BLASTN). Multiple sequence alignment was carried out; later, phylogenetic analysis was performed using software MEGA X (Altschul et al. 1990).

### Determination of MBL2 gene polymorphism

MBL2 gene exon 1 was amplified by direct PCR (Aydemir et al. 2011). PCR reaction mixtures (25 μl) contained 5 μl of the extracted DNA, Dream Tag Green PCR master mix(2X) 12.5 μl, 2 μl of each primer (0.5 μM), and water nuclease completed to 25 μl. The primers used for the reaction were 5′-TAGGACAGAGGGCATGCTC-3′ and reverse 5′CAGGCAGTTTCCTCTGGAAGG-3′. The PCR reactions were run for 40 cycles including 5 s at 98°C, 30 s at 58°C, and 30 s at 72°C with a final extension at 72°C for 1 min. PCR products were loaded directly into pits of 1.5% agarose gel and analyzed by electrophoresis. The PCR product sized 349 bp. The obtained PCR products were digested with restriction enzyme, Ban I, according to the manufacturer’s company protocol (BshNI; Thermo Scientific, USA). The restriction fragments were separated using a 2% agarose gel. Genotypes were determined as follows: genotype A/A (wild type) was two distinct products of 260 bp and 89 bp; genotype A/B (heterozygous variant allele) was three distinct products of 349 bp, 260 bp, and 89 bp; and genotype B/B (homozygous variant allele) was one 349 bp fragment. For MBL2 gene codon 54 polymorphisms, the normal allele is called A, and the variant allele is called B.

### Quantitation of serum MBL

MBL serum level was measured by sandwich enzyme-linked immunosorbent assay (ELISA) according to the manufacturer’s company protocol (Quantikine ELISA Human MBL; R&D Systems, Minneapolis, USA).

### Statistical analysis

The collected data were statistically analyzed using SPSS package version 21. Quantitative results were represented as mean ± standard deviation (SD), and qualitative results were expressed as number and percentages. The Kruskal-Wallis test was used for comparisons of the quantitative results with skewness. Chi-square was used to compare qualitative results. The relationships between the different variables were studied through correlation coefficient. The difference was considered significant at *P* value ≤ 0.05 levels.

## Results

**Fig. 1:**
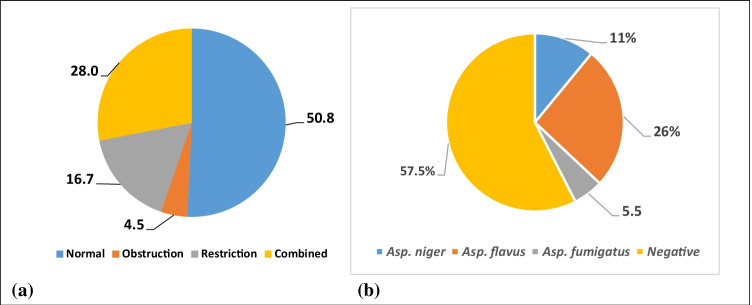
Percent of respiratory abnormalities according to PFTs (**a**). Percent of positive *Asp.* different strains according to sequencing (**b**)

Respiratory abnormalities according to the pulmonary function tests (PFTs) were found in 49.2% of workers, and the percent of different PFT diagnoses is illustrated in Fig. 1a, while blood sequencing for *Asp.* positivity revealed that 42.5% of the workers were positive for *Asp.* with different species, which is illustrated in Fig. 1b.

**Fig. 2:**
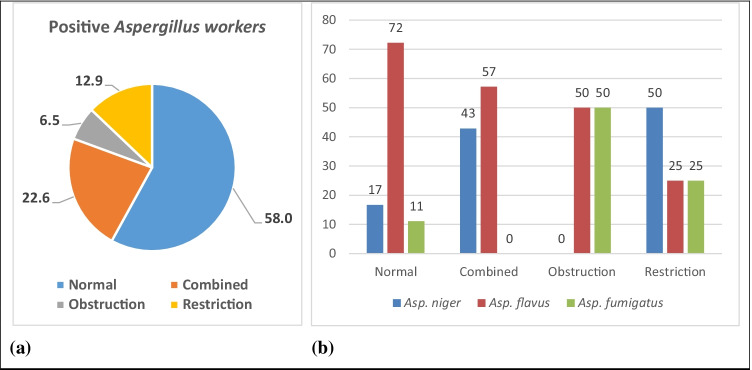
Prevalence of PFT abnormalities among the *Asp.*-positive workers (**a**), and the distribution of the workers according to abnormal PFTs according to the positivity to the different *Asp.* species (**b**)

Among the *Asp.*-positive workers, 6.5% of them showed obstruction, 12.9% revealed restriction, 22.6% were with combined PFT abnormalities, and 58% were normal (Fig. 2a)*.* Among workers with positive *Asp.*, 50% of the workers with obstructive PFTs were positive for *Asp. flavus* and 50% for *Asp. fumigatus*, while *Asp. flavus* was also found to be positive in 25% of workers with restrictive PFTs and 57% of those with combined abnormalities. *Asp. niger* was found to be positive in 50% of restrictive PFT workers, and about 43% of combined abnormal PFT workers (Fig. 2b). Thus, *Asp. niger* positivity was more common among workers with restrictive abnormalities. *Asp. fumigatus* was common in either restrictive or obstructive PFT abnormalities, but did not appear in those with combined PFT abnormalities.

**Fig. 3:**
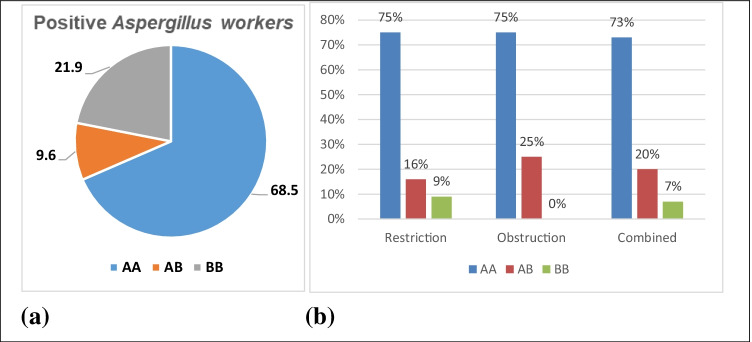
Percent of MBL2 genotypes among the workers with *Asp.*-positive sequencing (**a**), and among *Asp.*-positive workers with PFT abnormalities (**b**)

The wild genotype AA of the MBL2 gene was significantly common among *Asp.-*positive workers compared to the other genotypes (likelihood ratio= 9.86, *P* value = 0.02) (Fig. 3a), while there was no significant difference detected in the genotype of MBL2 among *Asp.*-positive workers with the different abnormal PFTs (likelihood ratio = 2.63, *P* value = 0.85) (Fig. 3b).

The levels of MBL were significantly lower among the workers with obstructive, restrictive, and combined restrictive-obstructive PFTs compared to those of workers with normal PFT, and those of the workers with restrictive PFTs compared to those with obstructive PFTs (Table 1). Moreover, MBL levels were significantly lower among the workers with positive *Asp.* compared to those with negative *Asp*., and MBL was significantly lower among the workers with positive *Asp. fumigatus* compared to those with positive *Asp. flavus* and *Asp. niger* (Table 1).

**Table 1:** Comparison of levels of serum MBL according to PFTs, and to *Aspergillus species*

	PFTs		*Aspergillus* sp.
	Mean ± SD		Mean ± SD
Obstruction	59.6±64.6 ^(N)^	*Asp. niger*	72.6±63.9 ^(neg)^
Restriction	16.67±2.08 ^(N, O)^	*Asp. flavus*	38.3±51.4 ^(neg)^
Combined	53.9±59.4 ^(N)^	*Asp. fumigatus*	15±20.6 ^(neg, nig, fl)^
Normal	101.1±62	Negative	127.1±54.7
*K-W*	9.95	*K-W*	10.74
*P* value	*P* = 0.02	*P* value	*P* = 0.013

*K-W*, Kruskal-Wallis test

*N*, significantly lower compared to normal; *O*, significantly lower compared to obstruction

*(neg)*, significantly lower than negative *Aspergillus*; *(fl)*, significantly lower than *Asp. flavus*; *(nig)*, significantly lower than *Asp. niger*

The current results showed that the *Asp.-*positive workers with homozygous or heterozygous A allele had significant decreased levels of serum MBL compared to the *Asp.-*positive workers with BB alleles, while there was no significant difference in the MBL levels in the different alleles among the *Asp.-*negative workers (Table 2).

**Table 2:** Comparisons of the MBL levels between the different MBL2 genotypes regarding *Aspergillus* positivity

		MBL (ng/ml)			
		Negative *Aspergillus*		Positive *Aspergillus*	
		Mean	±SD	Mean	±SD
MBL2 genotypes	AA	69.54	12.28	47.6(a)	8.71
	AB	66.78	30.25	21.1(a)	15.02
	BB	111.97	28.79	112.6	20.11
*K-W*		1.194		7.85	
*P* value		0.32		0.001	

*(a)*, significantly lower than in BB allele; *K-W*, Kruskal-Wallis test

## Discussion

*Aspergillus* (*Asp.*) is a fungal strain that can be found all over the world. Inhalation of airborne fungi could contribute to adverse health effects, mainly respiratory. Fungi may generate a broad variety of clinical syndromes. Asthma is a major health problem worldwide and is characterized by chronic airway inflammation after the interaction with allergens, environmental irritants, or infections (March et al. 2013). Early exposure to fungi is associated with increase asthma morbidity (Baxi et al. 2016).

In the final report of Saad-Hussein (2017)-2020 and in a previous study (Saad-Hussein et al. 2022), *Asp.* species from the air of the selected WWTP were isolated and identified under a microscope and through molecular sequencing of the air samples. *Asp.* with different strains were detected in the different areas of the selected WWTP, which increases the risk of developing occupational health hazards (Saad-Hussein 2017-2020; Saad-Hussein et al. 2022). Several studies recorded that WWTPs represent the most critical fungal contamination workplace (Wouters et al. 2006; WHO 2009). Therefore, the aim of the current study was the identification of the risk of *Asp.* positivity in workers exposed to different *Asp.* strains in their working environment of WWTP, and its potential hazards on their respiratory functions, and the geno-susceptible role of MBL2 gene polymorphism and serum MBL on the prognosis of *Asp.* positivity.

Diagnosis of fungal infections is complicated by the fact that sampling should be from the lower respiratory tract by bronchoalveolar lavage in order to culture the fungus, which could be a very harmful intervention and is mostly insensitive (Duthie and Denning 1995). In addition, Tarrand et al. (2003) denoted that blood culture is almost always negative for *Asp.* infections. Recent techniques in the form of molecular methods, such as polymerase chain reaction (PCR) and genomic sequencing, are used widely for fungal identification (Levetin 2004). Therefore, the lack of diagnostic tools for *Asp.* positivity led to the increasing importance of the non-culture diagnostic approaches, including PCR-based detection of *Asp.* DNA (Thornton 2008).

The main advantage of this detection technique is that unlike other detection methods, molecular sequencing has potentially very high sensitivity to detect *Asp.* signals. Furthermore, PCR methods for the detection of fungal DNA can also be tailored to detect all fungal species, or members of the genus *Asp.* or a particular *Asp.* species through specific designed primer and probes (Barton 2013).

So, in the present study we used both blood sequencing of the included workers to identify the positive workers for *Asp.* and PCR to identify the most common pathogenic *Asp.* species detected in the working environment of the selected WWTP. The results revealed that 26% of the workers were positive for *Asp. flavus*, 11% for *Asp. niger*, and 5.5% for *Asp. fumigatus.*

Many environmental fungi may increase the risk of pulmonary fungal infection that leads to hypersensitivity or complications in cases with lung diseases (Vicencio et al. 2010). The current study demonstrated an elevation of respiratory function abnormalities; approximately 5% of the workers showed obstructive abnormalities, 17% with restrictive PFTs, and 28% with combined restrictive-obstructive PFTs. These are consistent with the results of Cyprowski et al. (2015); they found that workers in WWTPs complained of respiratory symptoms and had impaired PFTs. The authors attributed these respiratory abnormalities to high exposure to environmental air pollutions and to high fungal load samples in air of WWTPs, as well as the presence of high amounts of dust particles where the fungal elements may be attached (WHO 2009; Cyprowski et al. 2015; Degois et al. 2017; Viegas et al. 2017; Saad-Hussein 2017-2020; Saad-Hussein et al. 2022).

The mechanism by which *Asp.* spores can invade human airways and pulmonary alveoli, causing a spectrum of diseases, is that it produces conidia of small size (2–3 μm) that is enough to reach the pulmonary alveoli, causing limitation of the pulmonary functions (Reponen et al. 1996; Latge 1999; Yaguchi 2011). In the present study, the PFTs of the examined workers revealed that abnormal PFT was detected in 42% of the workers with *Asp.* positivity; 6.5% of them had obstructive PFTs, 12.9% had restrictive PFTs, and 22.6% had combined restrictive-obstructive PFTs. It was known that obstructive lung disease results from narrowing of the smaller bronchi and larger bronchioles, which leads to difficulty in exhaling the air and ends by inflation of the lungs due to the abnormal high amount of air in the lungs, while restrictive lung diseases result from extra-pulmonary restriction that leads to restriction of lung expansion and decreased lung volume, and combined restrictive-obstructive lung disease occurs infrequently and is more commonly caused by a combination of pulmonary parenchymal and non-pulmonary disorders (American Thoracic Society 2019).

The abnormalities in the PFTs of the workers included in the present study were linked to the detection of specific species of *Asp.* with the different conidia sizes. Pulmonary functions of the workers with positive *Asp. flavus*, the most common species in the workers with positive *Asp.*, revealed that it was common among the workers with the three PFT abnormalities; as well as those with normal PFTs. The detection of *Asp. flavus*–positive workers more frequently among normal PFT workers could be attributed to that *Asp. flavus* is the second most common etiological agent of invasive aspergillosis, after *Asp. fumigatus* (Rudramurthy et al. 2019). But, due to its large size that decreases its possibility to penetrate to the distal bronchioles, it may be not essentially causing PFT impairments.

Although, the percent of workers positive to *Asp. fumigatus* and *Asp. flavus* in the current study was equal in the workers with obstructive PFTs, *Asp. fumigatus*–positive workers were detected in very few positive workers with normal PFTs. This could be attributed to that the conidia of *Asp. fumigatus*, with a diameter of approximately 1–4 μm, can be deposited in the lower respiratory tract causing obstructive PFTs, while *Asp. niger* and *Asp. flavus* were more common among the workers with restrictive as well as in the combined restrictive-obstructive PFT impairments. This could be because the larger sized conidia of *Asp. flavus* and *Asp. niger* tend to be deposited in the paranasal sinuses and upper airways as mentioned by Ben-Ami et al. (2010), which may explain the restrictive or combined restrictive-obstructive PFTs of those positive cases. Moreover, *Asp. niger* is less commonly considered a cause of invasive aspergillosis (Araiza et al. 2006), due to its large size that it cannot penetrate deep to the bronchioles and small bronchi. This could explain that there were no obstructive workers among *Asp. niger*–positive workers.

A previous research has found relationships between environmental fungal exposures and human health effects. The severity of the adverse health effects on the workers or exposed persons will also depend on their immune system (Tekaia and Latgé 2005)*.* Mannose-binding lectin (MBL) is recognized as an important host defense protein against fungal pathogens and affects innate immunity through activation of complement cascade via the antibody-independent pathway (Van Asbeck et al. 2008).

Although not all published studies have identified an association between MBL deficiency and the increase in the risk of fungal infections, many systemic fungal infective patients with low serum MBL levels were found to be at higher risk to develop severe complications (Pascale et al. 2013). The present results found that serum MBL levels could have a significant role on the hazardous complications of occupational exposure to *Asp.*, as the level of the serum MBL was significantly lower among the workers with obstructive and restrictive lung diseases compared to that in the workers with normal PFTs. Moreover, MBL was significantly lower in workers with restrictive PFTs compared to the workers with obstructive PFT impairments. This could be attributed to the development of pulmonary complications due to the MBL deficiency, as low MBL levels may lead to defective complement activation and decrease the host defense mechanism against fungal pathogens. Previously, Chong et al. (2005) suggested that MBL-mediated complement activation could provide immune complex for the removal of the pathogenic agents during infection. Therefore, low MBL levels may lead to defective complement activation and poor clearance immune complex activity against the fungal load in the exposed workers.

Moreover, the study revealed that serum MBL was significantly lower among the workers positive of the three *Aps.* species compared to the negative *Aps.,* as well as among workers with positive *Asp. fumigatus* compared to those with positive *Asp. flavus* and *Asp. niger*. This means that *Asp. fumigatus–*positive workers were associated with susceptibility to invasive infections and poor outcome due to their low MBL levels. This was similar to the results described by Eisen and Minchinton (2003). They mentioned that MBL binds bacteria, fungi, viruses, and parasite surfaces directly and indirectly by activation of lectin-complement pathway.

The MBL2 gene was found to influence the MBL level production in individuals (Eisen and Minchinton 2003). Regarding the MBL2 gene, the current study detected significant differences in the distribution of MBL2 genotype between workers with positive *Asp.*, as the wild genotype (AA) was significantly more dominant in the *Asp.-*positive workers (68.5%). This agrees with Aydemir et al. (2011); they reported that AA genotype was in 74% of the nosocomial fungi–infected patients and AB genotype in 26% and 30% of nosocomial patient and control groups, respectively. Moreover, there was a significant decrease in MBL levels detected among workers with homozygous or heterozygous allele A polymorphism among the *Asp.*-positive workers in the present study. These results are consistent with the findings of Garred et al. (2003); they found that serum MBL may be absent in individuals with homozygous or heterozygous A genotype.

For the PFTs of the workers with positive *Asp.*in the current study, about 75% of both the workers with obstructive PFTs and with restrictive PFTs and 73% of the workers with combined restrictive-obstructive PFTs had wild MBL2 genotype (AA), while mutant allele (AB) was found in about 25% of workers with obstructive, 16% in restrictive, and 20% in combined PFT workers, and none of the *Asp.-*positive workers with BB allele developed obstructive PFTs, and few developed restrictive or combined PFTs, 9% and 7% respectively.

Thus, in the present study, *Asp.*-positive workers with either homozygous or heterozygous wild allele A were at risk to develop obstructive or restrictive pulmonary function impairments. Few of the positive workers with homozygous B developed restrictive or combined PFTs, and none of BB genotype workers with positive *Asp.* developed obstructive PFTs. This means that homozygous B of MBL2 could have a protective role in minimizing respiratory function impairments secondary to positive *Asp.*, especially obstructive impairments. This could be due to the defense role of MBL, as serum MBL levels were significantly high in the *Asp.-*positive workers with homozygous B allele compared to those having homozygous or heterozygous A allele.

## Conclusions

Working in a WWTP can be associated with impaired PFTs due to exposure to airborne fungi. MBL2 genotyping showed that *Asp.-*positive workers with homozygous or heterozygous A allele were at risk to develop decline in their PFTs. Moreover, *Asp.-*positive workers with low MBL production are more prone to fungal positivity and decline in their pulmonary functions.

## Data Availability

Data sharing is not applicable to this article as no datasets were generated or analyzed during the current study.
